# Empirically informed symptom severity cutoffs for body dysmorphic disorder

**DOI:** 10.1016/j.jpsychires.2025.06.019

**Published:** 2025-06-17

**Authors:** David Mataix-Cols, Philip Andersson, Daniel Rautio, Oskar Flygare, Jennifer L. Greenberg, Susanne S. Hoeppner, Hilary Weingarden, Amita Jassi, Benedetta Monzani, Eric Hollander, David Castle, Georgina Krebs, Susan L. Rossell, Sabine Wilhelm, Christian Rück, Katharine A. Phillips, Lorena Fernández de la Cruz, Matti Cervin

**Affiliations:** a Center for Psychiatry Research, Department of Clinical Neuroscience, Karolinska Institutet & Stockholm Healthcare Services, Region Stockholm, Stockholm, Sweden; b Department of Clinical Sciences, Lund, Lund University, Sweden; c Massachusetts General Hospital and Harvard Medical School, Boston, USA; d National and Specialist OCD, BDD and Related Disorders Clinic for Young People, South London and Maudsley NHS Foundation Trust, London, UK; e Albert Einstein College of Medicine, Bronx, NY, USA; f University of Tasmania and Centre for Mental Health Service Innovation, Statewide Mental Health Service, Tasmania, Australia; g Research Department of Clinical, Educational & Health Psychology, University College London, UK; h Centre for Mental Health, School of Health Sciences, Swinburne University of Technology, Hawthorn, VIC, Australia; i Department of Mental Health, St Vincent’s Hospital, Melbourne, VIC, Australia; j Rhode Island Hospital and Alpert Medical School of Brown University, Providence, RI, USA; k New York-Presbyterian Hospital and Weill Cornell Medical College, New York, NY, USA

## Abstract

**Background:**

Symptom severity cutoffs for body dysmorphic disorder (BDD) are lacking, hindering communication between professionals and with the patient community.

**Method:**

We pooled data from 11 clinical trials or high-quality cohort studies from specialist clinics, totaling 804 individuals with BDD (80 % girls/women, 67 % adults). All participants had baseline scores on the adult or adolescent versions of the Yale-Brown Obsessive-Compulsive Scale Modified for BDD (BDD-YBOCS) and the Clinical Global Impressions–Severity scale (CGI-S). Receiver-operating characteristic analyses were used to identify BDD-YBOCS severity cutoffs, using the CGI-S as the benchmark measure. The classification performance of the cutoffs was evaluated in a holdout sample, consisting of 20 % of randomly selected participants.

**Results:**

No participants had subclinical symptoms and the cutoff for clinical versus subclinical cases was not computed. A BDD-YBOCS score ≥24 distinguished moderate from mild cases (area under the curve [AUC] = 0.72 [0.64–0.81]; accuracy: 77 %), a score ≥30 distinguished severe from moderate cases (AUC = 0.83 [0.90–0.87]; accuracy: 77 %), and a score ≥37 distinguished extreme from severe cases (AUC = 0.82 [0.77–0.87]; accuracy: 80 %). The classification performance of the cutoffs was modest in the holdout sample (62 %), but consistent cutoffs were found across sexes/genders, age groups (children and adults), and participants from Europe and the United States.

**Conclusion:**

These BDD-YBOCS severity cutoffs can be used for clinical and research purposes across different populations but should not be used as the sole basis for important clinical decisions affecting individual patients. The boundary between subclinical and clinical BDD will require further study.

## 1. Introduction

Body dysmorphic disorder (BDD) is a relatively prevalent, adolescent-onset mental disorder associated with substantial levels of distress and impairment (Rück et al., 2024). The Yale-Brown Obsessive-Compulsive Scale Modified for Body Dysmorphic Disorder (BDD-YBOCS) (Phillips et al., 1997, 2014) and its adolescent version (BDD-YBOCS-A)(Monzani et al., 2023) are universally used as the gold standard measure to quantify BDD symptom severity in both research trials and clinical practice. While the psychometric properties and sensitivity to change of both the adult and adolescent versions are well established (Monzani et al., 2023; Phillips et al., 1997, 2014), there is uncertainty regarding what scores correspond to different levels of BDD symptom severity, a fact that can hinder communication between professionals and with the patient community. For example, a BDD-YBOCS score of 23 could be interpreted as representing mild or moderate symptom severity, depending on who we ask. A score of 20 or 24 is typically used as the lower boundary for inclusion in clinical trials, but this is relatively arbitrary.

To our knowledge, no previous studies have attempted to calculate severity cutoffs for the BDD-YBOCS. To close this gap, we conducted a secondary analysis using pooled data from clinical trials and high-quality clinical cohort studies. To be clinically useful, such severity cutoffs should be invariant across sexes/genders, across the lifespan, and across individuals from different parts of the world. Therefore, we also examined the consistency of these cutoffs across different populations.

## 2. Methods

### 2.1. Participants and procedure

We contacted the corresponding authors of BDD clinical trials of psychological and pharmacological treatments from around the world and invited them to contribute their individual participant data for a secondary analysis. From the same authors, we also requested data from patient cohorts collected in their specialist clinics. To be eligible, the datasets had to include strictly diagnosed BDD cases (either though semi-structured diagnostic interviews or multidisciplinary assessments done in specialized BDD clinics) and have the relevant data available at baseline, that is, the BDD-YBOCS (or BDD-YBOCS-A) and the Clinical Global Impressions–Severity scale (CGI-S). We also collected information on sex or gender (as originally recorded in each individual study) and age of the individual participants.

We received 11 different datasets corresponding to four randomized controlled trials (RCTs) (Mataix-Cols et al., 2015; Rossell, 2022; Wilhelm et al., 2014, 2022), three open trials (Gentile et al., 2019; Phillips et al., 2016; Rautio et al., 2023), and three clinical cohorts from specialist clinics (Lundström et al., 2023; Rautio et al., 2022a, 2022b). One dataset (Rossell, 2022) was from a clinical trial yet to be published. Seven datasets included data from adults and four from children and adolescents. Individuals in each individual dataset who had missing data on the baseline BDD-YBOCS (or BDD-YBOCS-A in the studies including young people) or the CGI-S were excluded. Preliminary exploration of the data revealed that the sample only included one subclinical case (BDD-YBOCS score = 11; CGI-S score <3), which was excluded, resulting in a final dataset of 804 participants for analysis. Characteristics of the individual datasets are described in Table 1, and a summary of the demographic and clinical characteristics of the whole sample and subsamples is included in Table 2.

Participants from all the clinical trials (except for those from the Wilhelm et al., 2022 study) had a minimum score of 20 or 24 on the BDD-YBOCS (or BDD-YBOCS-A), which was an inclusion requirement for each of the trials (Table 1). Individuals from the specialist clinics did not have a minimum score requirement but met diagnostic criteria for BDD at baseline.

Each individual study was approved by the corresponding institutional/ethical review board or audit committee in each country. All participants provided written informed consent (and assent if under the age of 18) for participation, except for the participants in the clinical cohort from the London pediatric specialist clinic, where informed consent was not required because the study was part of an audit of routinely collected clinical data. Further details regarding the ethical approval/permits for each of the samples can be found in the original studies (Enander et al., 2016; Gentile et al., 2019; Lundström et al., 2023; Mataix-Cols et al., 2015; Phillips et al., 2016; Rautio et al., 2022a, 2022b, 2023; Rossell, 2022; Wilhelm et al., 2014, 2022).

### 2.2. Measures

#### Body dysmorphic disorder diagnosis.

For all participants, a current diagnosis of BDD was made in accordance with the diagnostic criteria from the Diagnostic and Statistical Manual of Mental Disorders, fourth edition, Text Revised (DSM-IV-TR) or fifth edition (DSM-5) by means of a clinical interview by expert clinicians or trained raters supported by semi-structured interviews (Enander et al., 2016; Gentile et al., 2019; Lundström et al., 2023; Mataix-Cols et al., 2015; Phillips et al., 2016; Rautio et al., 2023; Rautio et al., 2022a; Rautio et al., 2022b; Rossell, 2022; Wilhelm et al., 2014; Wilhelm et al., 2022) (Table 1).

#### Yale-Brown Obsessive-Compulsive Scale Modified for Body Dysmorphic Disorder.

The BDD-YBOCS (or its adolescent version, the BDD-YBOCS-A) is a 12-item, clinician- or rater-administered scale that assesses BDD symptom severity during the past week. It is used to assess current BDD severity in individuals who have already been diagnosed with BDD based on a clinical interview or a structured diagnostic instrument. The first five items of the BDD-YBOCS assess obsessional preoccupations about perceived appearance defects, items six to 10 assess BDD-related repetitive behaviors (i.e., compulsions, rituals), item 11 assesses insight regarding appearance beliefs, and item 12 assesses avoidance of life activities. Each item is scored on a Likert scale ranging from 0 to 4, with the total score ranging from 0 to 48. Higher scores reflect more severe symptoms. Both the adult and adolescent versions have strong interrater and test-retest reliability, internal consistency, validity, and sensitivity to change (Monzani et al., 2023; Phillips et al., 1997, 2014). While factor analytical studies of the BDD-YBOCS items have inconsistently revealed at least two factors (e.g., Kollei et al., 2023; Monzani et al., 2023; Phillips et al., 2014), in practice, only the total score is used both in clinical practice and research.

#### Clinical Global Impressions – Severity scale.

The CGI-S is a single-item measure where the clinician or rater makes an overall severity rating of a specific disorder accounting for all available information about the patient, including but not limited to current symptoms, impairment, and general functioning (Busner and Targum, 2007; NIMH, 1976). In this study, CGI-S ratings were made in relation to BDD, regardless of any potential comorbidities. The CGI-S is scored on a scale from 1 to 7. In the present study, following the CGI-S score descriptions (Busner and Targum, 2007; Rosen et al., 1984), scores of 1 (“normal, not at all ill”) and 2 (“borderline mentally ill”) were grouped to reflect subclinical BDD, scores of 3 (“mildly ill”) were selected to reflect mild BDD symptom severity, scores of 4 (“moderately ill”) were selected to reflect *moderate BDD symptom severity*, scores of 5 (“markedly ill”) were selected to reflect *severe BDD symptom severity*, and scores of 6 (“severely ill”) and 7 (“among the most extremely ill patients”) were grouped to reflect *extreme BDD symptom severity*.

### 2.3. Statistical analysis

All statistical analyses were performed in R Studio. We examined the association between the BDD-YBOCS/BDD-YBOCS-A (hereafter referred to as BDD-YBOCS only) and the severity groupings according to CGI-S using Spearman’s rho and an ordinal regression model with BDD-YBOCS symptom severity score as the independent variable and CGI-S severity group as the dependent variable.

To identify the optimal cutoffs, we used receiver-operating characteristic (ROC) analyses implemented in the *pROC* and *cutpointr* libraries. The area under the curve (AUC) values were used to evaluate the ability of the BDD-YBOCS to distinguish between the different CGI-S severity groups. AUC values from 0.7 to 0.8 were considered acceptable, values from 0.8 to 0.9 were considered excellent, and values above 0.9 were considered outstanding. Both the mild and extreme groups had comparably few participants, revealing a potential sampling bias. The mild group also had a left-skewed distribution (Skewness = – 0.55). Unbalanced and skewed groups create difficulties when establishing cutoffs, as larger groups will be prioritized over smaller groups if accuracy measures are used. Conversely, if measures aimed at maximizing the sum of sensitivity and specificity are used, smaller groups exert too much dominance, leading to decreases in accuracy, particularly when the small groups are skewed as in the present case. To respect the potential sampling bias and skewness, cutoffs in relation to the smaller groups were established by maximizing sensitivity in relation to the larger group. Thus, when the cutoff between the mild and moderate groups was estimated, we aimed to maximize sensitivity for the larger moderate groups while constraining specificity for the smaller mild group to not be lower than 0.4. Similarly, when the cutoff between the severe and extreme groups was estimated, we aimed to maximize sensitivity for the larger severe group while constraining specificity for the smaller extreme group to not be lower than 0.5. The 0.4 and 0.5 values were based on the size of the group, with the extreme group being proportionally larger than the mild group. This approach balances accuracy with the ability to distinguish smaller groups. When identifying the cutoff between moderate and severe groups, which were balanced, we used the Youden index, which maximizes the sum of sensitivity and specificity.

For the analyses, we first used the *rsample* package to reproducibly split (using a predefined seed) the full sample into an exploratory sample (80 % of participants), used to identify empirically informed cutoffs, and a holdout sample (20 % of participants), used to evaluate the classification performance of the cutoffs. Classification performance was evaluated using AUC values in the exploratory sample and the overall accuracy of the cutoffs in classifying participants in the holdout sample into the mild, moderate, severe, and extreme symptom severity categories based on their BDD-YBOCS scores. Last, to explore the consistency of the cutoffs, we identified cutoffs separately by age group (children/adolescents and adults), sex/gender (girls/women and boys/men; other genders were excluded due to low numbers), and continent (American and European participants; participants in one global study (Gentile et al., 2019) and Australian trial (Rossell, 2022) were excluded due to small numbers.

Regarding statistical power, to identify an AUC of at least 0.75 in balanced groups with a power of 0.80 and an alpha level of 0.05, a total sample of 251 participants would be needed (Hanley and McNeil, 1982). Thus, for a majority of the AUC analyses, we had sufficient power, while we were underpowered for some subgroup analyses, particularly those that included the mild and extreme symptom severity groups.

## 3. Results

### 3.1. Descriptive statistics

We first examined the number of participants in each severity group according to the benchmark measure, the CGI-S. The largest group was the moderate symptom severity group (*n* = 330, 41 %), followed by the severe symptom severity group (*n* = 305, 38 %), the extreme symptom severity group (*n* = 125, 16 %), and the mild symptom severity group (*n* = 44, 6 %). There were no participants in the dataset with CGI-S scores of 1 (“normal, not at all ill”) or 2 (“borderline mentally ill”) corresponding to subclinical BDD.

Spearman’s rho indicated that BDD-YBOCS and CGI-S severity group correlated strongly (*r* = 0.71, *p* < 0.001, ranging from 0.44 to 0.79 across individual studies). An ordinal regression model with the BDD-YBOCS score as the independent variable and the CGI-S severity groups as the dependent variable was statistically significant (*p* < 0.001). The Nagelkerke’s pseudo *R*^*2*^ estimate of the model indicated that variation in BDD-YBOCS severity accounted for 54 % of the variation in the CGI-S severity classification.

### 3.2. Estimating and evaluating cutoffs

As there were no participants classed as subclinical in the final dataset, we could not calculate cutoffs to distinguish sub-clinical from mildly ill cases.

The BDD-YBOCS acceptably distinguished between mild and moderate cases (AUC = 0.73 [95 % CI, 0.64–0.81]). The optimal cutoff suggested that moderate severity started at a BDD-YBOCS score of 24. This score yielded an accuracy of 77 %, a sensitivity of 82 %, and a specificity of 44 %. That is, 82 % of the moderate cases and 44 % of the mild cases were correctly classified.

The BDD-YBOCS excellently distinguished between moderate and severe cases (AUC = 0.83 [95 % CI, 0.80–0.87]). The optimal cutoff suggested that the group corresponding to severe BDD started at a BDD-YBOCS score of 30. This score yielded an accuracy of 77 %, a sensitivity of 78 %, and a specificity of 77 %.

The BDD-YBOCS also excellently distinguished between severe and extreme BDD cases (AUC = 0.82 [95 % CI, 0.77–0.87]). The optimal cutoff suggested that the extreme class started at a BDD-YBOCS score of 37 points. This score yielded an accuracy of 80 %, a sensitivity of 89 %, and a specificity of 60 %.

### 3.3. Holdout sample and subgroup analyses

When classifying participants in the holdout sample (the remaining 20 % of participants) into severity groups using the above cutoffs, the precise classification performance was 62 %. Among true mild cases, 50 % were correctly classified, and among true moderate, severe, and extreme cases, 58 %, 71 %, and 52 % were correctly classified. No true mild cases were classified as extreme, and no true extreme cases were classified as mild. To further explore misclassification, we examined each misclassified case and assessed how far it deviated from its correct category. A large majority (88 %) of misclassifications appeared in either one severity category above or below the “true” category.

Finally, the resulting cutoffs were remarkably consistent across subgroups based on sex/gender, age group, and continent (Table 3). In a post-hoc analysis, we also examined cutoffs separately in studies requiring versus not requiring a minimum entry score on the BDD-YBOCS. Almost identical cutoffs as in the full sample emerged for distinguishing moderate from severe and severe from extreme cases. However, the cutoff for distinguishing moderate from mild cases was lower in studies not requiring a minimum score for entry (Table 3).

## 4. Discussion

This secondary analysis aimed to address the lack of clear guidance on how scores on the BDD-YBOCS translate into meaningful and easily communicable severity groups. Using empirical guidance, we suggest that BDD-YBOCS scores of 24–29 correspond to moderate BDD cases, scores from 30 to 36 to severe BDD cases, and scores from 37 to 48 points to extreme BDD cases. Our results also suggest that these cutoffs generally apply across sexes/genders, age groups, and continents. These empirically derived severity cutoffs are congruent with our clinical experience with this scale.

As there were no cases with subclinical symptoms in our dataset (i.e., CGI-S scores of 1 or 2), we could not calculate the BDD-YBOCS cutoff differentiating subclinical from clinical cases. While we could establish that scores <24 correspond to mild BDD, the lower boundary for mild BDD is still unknown. This will need to be addressed in future studies including individuals with a wider range of symptom severities recruited from the community. Thus, researchers and clinicians should not assume that patients with scores <24 do not have a diagnosis of BDD and do not need treatment. In fact, several of the included clinical trials had entry requirements of at least 20 points.

Using a holdout sample, we examined the degree to which our derived severity cutoffs could classify individuals into the correct group of BDD symptom severity. The classification performance was modest, ranging from 50 % to 71 % across severity groups. This was somewhat expected given that symptom severity is a continuous rather than categorical construct and most misclassifications appeared close to the derived cutoffs. Further, the observed distribution of the data, which was largely concentrated in the moderate-to-severe range, introduced difficulties in achieving high accuracy while, at the same time, distinguishing the smaller groups. Furthermore, the milder and extreme severity groups were skewed, indicating that the suggested cutoffs should work better in more diverse populations, where a larger proportion of patients are expected to have mild and extreme BDD. In light of modest accuracy, we caution against the exclusive use of these cutoffs to guide important clinical decisions or resource allocation regarding individual patients. Other relevant variables should be used, together with BDD-YBOCS scores, such as duration of the disorder, time without adequate treatment, psychiatric and somatic comorbidities, family accommodation, socioeconomic circumstances, personal treatment history, and patient preferences and values, to name a few.

One strength of this study was the broad representation of individuals of all ages from several countries. The data were of high quality and collected by expert raters. The main limitation was the moderate variability in the data, particularly at the lower and higher ends of the distribution. Very few participants with mild and extreme BDD were included, resulting in difficulties with identifying the optimal cutoffs and modest accuracy for these categories. When splitting the sample according to whether or not the study required a minimum BDD-YBOCS score at entry, the mild-BDD cutoff was lower in studies without such requirements, highlighting how sample composition can affect estimates. However, these analyses may have been underpowered. Future studies should examine the validity of the resulting cutoffs vis-à-vis external validators such as self-rated BDD symptoms or measures of BDD-related functional impairment. Despite our best efforts to include all available datasets worldwide, we could not include a number of relevant studies because they did not use the CGI-S in their assessment batteries. The single-item CGI-S also has limitations. To our knowledge, its inter-reliability has not been explored in BDD. Furthermore, as both the BDD-YBOCS and the GGI-S are typically scored by the same raters, it may have resulted in inflated correlations. However, we found that these measures were only moderately correlated in our dataset, sharing 54 % of the variance, which suggests that the instruments do not capture exactly the same phenomena. Indeed, the CGI-S likely captures a broader range of difficulties not captured by the BDD-YBOCS. Statistical power was limited for some of the subgroup analyses but sufficient for our main analyses. Another limitation was that we did not collect information on ethnicity, which precluded analyses based on further subgroups.

To conclude, we provide the field with empirically informed BDD-YBOCS severity cutoffs across the lifespan, which will hopefully be useful in research and clinical settings. Further study of the boundary between subclinical and clinical BDD is needed.

## Figures and Tables

**Table 1:** Characteristics of the datasets included in the study, totaling 804 included individuals.

Study	Type of study	Country	Age group	Original study sample size	Sample size used in the current study	Assessment of BDD diagnosis	Required minimum BDD-YBOCS/BDD-YBOCS-A score at baseline
Wilhelm et al. (2014)	RCT	United States	Adults	36	35	SCID-I	≥24
Mataix-Cols et al. (2015)	RCT	England	Children (12–18 years)	30	30	SCID-I	≥24
Phillips et al. (2016) ^a^	Open trial	United States	Adults	100	99	SCID-I	≥24
Enander et al. (2016)	RCT	Sweden	Adults	94	94	SCID-I	≥20
Gentile et al. (2019)	Open trial	Global^b^	Adults	34	31	SCID-5	≥20
Wilhelm et al. (2022)	RCT	United States	Adults	80	80	MINI 7.02, with additional BDD module	Not indicated (lowest score in dataset 18)
Lundström et al. (2023)	Clinical cohort	Sweden	Adults	163	137	SCID-I	NA (lowest score in dataset 12)
Rautio et al. (2023)	Open trial	Sweden	Children (12–17 years)	19	19	SCID-I, adapted to align with DSM-5 criteria	≥24
Rautio et al. (2022a, 2022b)^c^ [Stockholm sample]	Clinical cohort	Sweden	Children (10–17 years)	NA	154	SCID-I, adapted to align with DSM-5 criteria	NA (lowest score in dataset 18)
Rautio et al. (2022a, 2022b)^c^ [London sample]	Clinical cohort	England	Children (13–17 years)	NA	64	DAWBA	NA (lowest score in dataset 24)
Rossell (2022) [unpublished]^d^	RCT	Australia	Adults	62	61	SCID-5	≥20 (lowest score in the whole dataset was 11; lowest score was 16 after excluding a participant with a CGI-S score <3)^e^

*Abbreviations:* BDD, body dysmorphic disorder; BDD-YBOCS, Yale-Brown Obsessive-Compulsive Scale Modified for Body Dysmorphic Disorder; BDD-YBOCS-A, BDD-YBOCS, adolescent version; DAWBA, Development and Well-Being Assessment; CGI-S, Clinical Global Impression - Severity; MINI, Mini International Neuropsychiatric Interview; NA, not applicable; RCT, randomized controlled trial; SCID-I, Structured Clinical Interview for DSM-IV axis I disorders; SCID-5, Structured Clinical Interview for DSM-5.

a This relapse prevention study consisted of an initial open-label phase followed by a second phase that consisted of a randomized controlled trial. Data from the first phase were used in the present report.

b This study was internet-based and the sample included participants from 9 countries and 12 different nationalities, including American, Swedish, Indian, Bulgarian, Finnish, English, Serbian, South Korean, English, Norwegian, Sri Lankan, and Lithuanian.

c These cohort studies are still actively recruiting and the number of included participants is larger than that of the cited references.

d This study is unpublished, but characteristics of the study and the sample can be found in the cited trial registration.

e ≥ 20 was required at screening which was up to four weeks before the baseline assessment (i.e., baseline assessments may present with scores lower than 20, but BDD criteria were still met).

**Table 2:** Demographic and clinical characteristics of the sample (N = 804).

Variable	
Demographic characteristics	
*Age*, mean years (sd)	
Total sample	25.7 (11.6)
Children	15.6 (1.4)
Adults	31.3 (10.9)
***Age group*, n (%)**	
Children	265 (33 %)
Adults	540 (67 %)
***Sex/gender*, n (%)**	
Girls/women	647 (80 %)
Boys/men	156 (19 %)
Other	2 (0.2 %)
***Country*, n (%)**	
Sweden	404 (50 %)
United States	214 (27 %)
England	94 (12 %)
Australia	61 (8 %)
Global	31 (4 %)
**Baseline BDD symptom severity**	
***BDD-YBOCS or BDD-YBOCS-A score*, mean (sd)**	
Total sample	30.3 (5.7)
*By age group*	
Children	31.4 (5.7)
Adults	29.8 (5.7)
*By gender*	
Girls/women	30.1 (5.5)
Boys/men	30.4 (5.8)
Other	27.0 (1.4)
*By continent*	
Europe (Sweden + England)	29.9 (5.9)
America (United States)	31.8 (4.9)
Oceania (Australia)	30.0 (6.7)
Global^a^	27.9 (5.0)
***CGI-S score*, n (%)**	
1. Normal, not at all ill	0 (0 %)
2. Borderline mentally ill	0 (0 %)
3. Mildly ill	44 (6 %)
4. Moderately ill	330 (41 %)
5. Markedly ill	305 (38 %)
6. Severely ill	112 (14 %)
7. Among the most extremely ill patients	13 (2 %)

*Abbreviations:* BDD-YBOCS, Yale-Brown Obsessive-Compulsive Scale Modified for Body Dysmorphic Disorder; BDD-YBOCS-A, Yale-Brown Obsessive-Compulsive Scale Modified for Body Dysmorphic Disorder, adolescent version; CGI-S, Clinical Global Impressions – Severity.

a This study was internet-based and the sample included participants from 9 countries and 12 different nationalities, including American, Swedish, Indian, Bulgarian, Finnish, English, Serbian, South Korean, English, Norwegian, Sri Lankan, and Lithuanian.

**Table 3:** Estimated severity cutoffs for specific subsamples.

	Moderate BDD symptom severity corresponds to scores ≥	Severe BDD symptom severity corresponds to scores ≥	Extreme BDD symptom severity corresponds to scores ≥
Cutoffs from the exploratory sample	24	30	37
*Cutoffs by age group*			
Children/adolescents	25	30	38
Adults	23	30	37
*Cutoffs by sex/gender*			
Girls/women	23	29	37
Boys/men	25	30	37
*Cutoffs by continent*			
Europe (Sweden + England)	23	30	37
America (United States)	23	30	37
*Cutoffs by required minimum score at entry*			
No required minimum score	22	30	37
A required minimum score ≥ 20	26	30	38

*Abbreviations:* BDD, body dysmorphic disorder.
